# Validation of maternal report of nutrition‐related interventions and counselling during antenatal care in southern Nepal

**DOI:** 10.1111/mcn.13303

**Published:** 2021-12-14

**Authors:** Emily Bryce, Joanne Katz, Rebecca Heidkamp, Tsering Pema Lama, Subarna K. Khatry, Steve LeClerq, Melinda Munos

**Affiliations:** ^1^ Department of International Health Johns Hopkins Bloomberg School of Public Health Baltimore Maryland USA; ^2^ Nepal Nutrition Intervention Project, Sarlahi (NNIPS) Kathmandu Nepal

**Keywords:** area under curve, counselling, mental recall, pregnancy, prenatal care, sensitivity and specificity, surveys and questionnaires

## Abstract

The delivery of nutrition‐related interventions and counselling during antenatal care is critical for a healthy pregnancy for both mother and child. However, the accuracy of maternal reports of many of these services during household surveys has not yet been examined. Our objectives were to assess the validity of the maternal reports of 10 antenatal nutrition interventions, including counselling, and examine associates between maternal characteristics and accuracy. Maternal report of services received collected during a post‐partum survey was compared to the gold standard, the direct observation of all women's antenatal care visits. Individual‐level validity was assessed by calculating indicator sensitivity, specificity and area under the operating curve (AUC). The inflation factor (IF) measured population‐level bias. For five indicators, the high true coverage limited our ability to assess the validity of the maternal reports. There were no indicators that had both high individual‐level validity (AUC > 0.70) and low population bias (0.75 < IF < 1.25). Indicators with greater true coverage estimates had higher sensitivity and lower specificity estimates compared to those indicators with lower true coverage. There were no maternal characteristics associated with the accuracy of the report. Maternal report of antenatal nutrition‐related interventions and counselling during household surveys was found to have variable validity across indicators. Additional research in settings with varying coverage levels should be considered to best inform antenatal care coverage measurement in household surveys.

## Key Messages

- The coverage of nutrition‐related interventions and counselling was high (>89%) for the majority of indicators.
- Maternal reports resulted in higher coverage estimates than what was observed for all but one indicator.
- No indicator assessed had high individual level validity (AUC > 0.70) and low population bias (0.75 < IF < 1.25).
- The greater the indicator's true coverage estimate, the greater the sensitivity and lower the specificity values.

## Graphical abstract

Our objectives were to assess the validity of the maternal reports of 10 antenatal nutrition interventions, including counselling, and examine associates between maternal characteristics and accuracy. There were no indicators that had both high individual‐level validity (AUC > 0.70) and low population bias (0.75 < IF < 1.25).

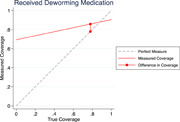

## 1 INTRODUCTION

Adequate nutrition during pregnancy is vital for the health of both mothers and infants. Poor quality of diet, including lack of diversity of foods consumed and low caloric intake, are two routes of experiencing malnutrition during pregnancy, although the two are not mutually exclusive. Multiple nutrient deficiencies, such as iron and calcium, have been associated with poor maternal and infant outcomes, including pregnancy‐induced hypertension, preterm birth, low birth weight and death (Black et al., 2008, 2013; Christian et al., 2008). Low maternal body mass index (BMI), defined as <18.5 kg/m^2^ and inadequate maternal weight gain have also been linked to poor birth outcomes (Black et al., 2013; Christian et al., 2008; Han, Mulla, et al., 2011; Han, Lutsiv, et al., 2011). Furthermore, it has been estimated that maternal undernutrition contributes to 800,000 neonatal deaths each year (Bhutta et al., 2013).

Micronutrient supplementation, deworming and counselling on diet and healthy behaviours during pregnancy are commonly delivered through antenatal care (ANC) to improve health and nutrition outcomes in both mothers and infants (Bhutta et al., 2008; WHO, 2016). Calcium supplementation has been shown to reduce preterm birth in women with low calcium intake and incidence of pre‐eclampsia and eclampsia, which are a leading cause of maternal mortality (Hofmeyr et al., 2018; Say et al., 2014). Mass deworming during pregnancy reduces maternal anaemia by 23%, although it had no effect on birth outcomes (Salam et al., 2019). Nutrition education and counselling (NEC) commonly provide information on increasing nutrient intake during pregnancy, with an emphasis on protein and micronutrients, use of fortified foods and/or adherence to supplements (WHO, 2016; Girard & Olude, 2012). NEC can increase energy and protein intake, gestational weight gain and birth weight and decrease the risk of anaemia and preterm birth (Girard & Olude, 2012; Ota et al., 2015; Nikièma et al., 2017; Demilew et al., 2020). Gestational weight gain that does not comply with National Academy of Medicine guidelines is associated with poor birth outcomes; small for gestational age births and preterm birth for weight gain below recommended levels and large for gestational age, macrosomia and caesarean delivery for weight gain above recommended levels (Goldstein et al., 2017). NEC can also improve adherence to supplementation; in a study in Nepal, when NEC was included with iron folic‐acid supplementation compliance and maternal haemoglobin levels were improved compared to women receiving supplementation only (Adhikari et al., 2009). Separate from NEC, counselling about risks of tobacco and alcohol use during pregnancy (WHO, 2016), including reduced birth weight and size, is also recommended (Abraham et al., 2017; Nykjaer et al., 2014).

In many low‐and‐middle‐income countries (LMIC) including Nepal, large population‐based surveys are used to collect data on intervention coverage including ANC services. The Demographic Health Survey (DHS) Program has conducted over 400 surveys in more than 90 countries since 1984 (The DHS Program, 2021). However, many of the indicators for nutrition‐related interventions and counselling during ANC have not yet been validated to determine if maternal report produces accurate data. In the DHS, data on ANC are collected by asking women with a live birth in the last 5 years (starting with DHS8, 3 years) to recall the interventions she received during pregnancy. It is necessary that the current coverage of these interventions is accurately assessed to identify the populations in need, to track progress in improving coverage, and to plan future health programming accordingly. The DHS program revises the questionnaires every 5 years, which offers an opportunity to add, drop or improve questions based on newly generated evidence.

- The coverage of nutrition‐related interventions and counselling was high (>89%) for the majority of indicators.
- Maternal reports resulted in higher coverage estimates than what was observed for all but one indicator.
- No indicator assessed had high individual level validity (AUC > 0.70) and low population bias (0.75 < IF < 1.25).
- The greater the indicator's true coverage estimate, the greater the sensitivity and lower the specificity values.

Previous validation studies examining indicators of ANC, labour and delivery, post‐natal or care‐seeking for child illness have demonstrated that the accuracy of answers obtained by maternal recall is varied (Blanc et al., 2016; Carter et al., 2018; McCarthy et al., 2016, 2020) One study in China examined maternal recall of ANC compared to medical records, however, this study did not examine the validity of nutrition services, apart from weight measurement, or NEC, only family planning advice (Liu et al., 2013). Another study examining indicators of ANC in Bangladesh, Cambodia and Kenya found higher validity for observable actions, such as weight measurement than counselling (McCarthy et al., 2020). That study compared direct observation to maternal report at an exit interview immediately following the observation. A study in the same study area in Sarlahi, Nepal, assessed examination of maternal recall of birthweight to identify low birth weight infants. The authors reported low sensitivity for maternal recall of this information (Chang et al., 2018).

This study aimed to assess the validity of the maternal reports of receipt of antenatal nutrition‐related interventions and NEC. A secondary aim was to examine any maternal characteristics associated with the accurate recall of the nutrition‐related interventions and NEC.

## 2 METHODS

### 2.1 Study sites and participants

The study population included pregnant women who presented for their first ANC visit at one of five public health posts in two municipalities in Sarlahi district Nepal between December 2018 and November 2019. Sites were within the Nepal Nutrition Intervention Project Sarlahi (NNIPS) study area which is located in Nepal's Province 2. The sites were chosen based on ANC client caseload, accessibility and were limited to two municipalities for approval purposes. This Province has the greatest proportion of women with a low BMI (<18.5 0 kg/m^2^), short height (<145 cm) and anaemia (<11.0 g/dl for pregnant women, <12.0 g/dl for nonpregnant women) of all seven provinces, indicating a great need for nutrition interventions (Nepal MoH, 2017). The Sarlahi district is located in the Southern Terai region, bordering the Indian state of Bihar. Women were considered eligible if they were married, 15 years old or older, lived in the study area at the time of enrolment and did not plan to leave the study area during the study period. Women who had already received ANC care for this pregnancy received an ultrasound scan during this pregnancy or were planning to leave the study area were deemed ineligible.

The target sample size of 300 women for the validation study was established using the estimated iron folic‐acid coverage of 50% from the 2016 DHS, as this was one of the primary indicators of interest (Bryce et al., 2021; Nepal MoH, 2017). This would allow for a 0.13 wide 95% confidence interval around an area under the curve (AUC) estimate of 0.50 (Munos et al., 2018). The study aimed to enrol 450 women to account for loss to follow‐up, including women who sought ANC elsewhere or did not have a live birth.

### 2.2 Data collection

Direct observation of ANC visits was used to establish the gold standard for the validation of maternal reports of nutrition‐related interventions and counselling (Munos et al., 2018). Trained study staff observed all ANC visits the enrolled participants attended at one or more of the five health posts. Before study initiation, the data collector training included didactic instruction and training on the checklist via prerecorded videos of mock ANC visits. Additionally, the data collectors observed real ANC visits using the 28‐item checklist, which was then compared to the trainer's record of the visit using the same checklist. Data collectors used a checklist of 28 items to record whether a specific intervention was ‘provided’ or ‘not provided’. Study staff also administered a short demographic questionnaire at the enrolment and a brief follow‐up questionnaire at each subsequent ANC visit. The follow‐up questionnaire inquired about care‐seeking between observed visits. The specific question asked was ‘Since the last time NNIPS staff talked to you up until now, have you received advice from any health providers about nutrition for your pregnancy?’. This was done to attempt to improve the gold standard by identifying a subset of participants where all interventions provided were observed by the study team. Post‐partum interviews to assess the maternal reports of interventions received were conducted on average 6 months after delivery at the woman‘s home or her familial home, called her *maiti*. During this interview, data were also collected on socioeconomic status and pregnancy outcome. Following the interview, the study staff noted whether there was anyone else (i.e., husband, mother‐in‐law) present during the interview and whether the study staff felt the individual helped answer the questions ‘never, a little or a lot’.

Enrolment was completed by November 2019 and direct observation of subsequent ANC visits continued through mid‐March 2020 when all nonemergency health services were disrupted by the COVID‐19 pandemic. The COVID‐19 shutdown delayed a portion of the post‐partum interviews, resulting in some recall periods greater than 6 months. There were 26 women that had not yet delivered at the time of the shutdown. Given that only emergency services were still offered at the health posts, we do not believe that we missed any routine ANC. The post‐partum interviews resumed on 8 May  2020, but were halted again 5 days later (18 were completed during this time). The post‐partum interviews were resumed on 8 August 2020, were completed in November 2020.

### 2.3 Analysis

The sensitivity (Se) and specificity (Sp) were calculated from 2 × 2 tables comparing the gold standard direct observation during ANC to the post‐partum maternal report of interventions provided during ANC with 95% confidence intervals assuming a binomial distribution. The calculations based on a small number of true positives or true negatives are presented but flagged to interpret with caution. This is because these estimates have a high degree of uncertainty (95% confidence intervals greater than 15 percentage points) ( McCarthy et al., 2020). The area under the operating curve (AUC) is typically used to compare cut‐offs for diagnostic tests by plotting the sensitivity against 1‐specificity, but in this case, it represents a summary measure of individual‐level validity. An AUC equal to 0.50 represents an indicator performing as well as a random guess and an AUC equal to 1 represents perfect validity (Munos et al., 2018). Previous validation studies have used different AUC cut‐offs to represent high individual‐level validity, for example, AUC ≥ 0.60, AUC ≥ 0.67 or AUC ≥ 0.70 (Chang et al., 2018; Liu et al., 2013; McCarthy et al., 2018; Stanton et al., 2013), and for this study, we determined a priori a cut of AUC ≥ 0.70 as high individual‐level validity (Munos et al., 2018).

The population‐level validity of the indicators was assessed by estimating the inflation factor (IF). The IF represents whether the indicator coverage measured by the survey would be over or underestimated in the setting, calculated by dividing the study coverage (Pr) by the true coverage (*P*). The true coverage is from the gold standard direct observation. The study coverage is calculated using the indicator sensitivity and specificity in the following equation: Pr = *P* × (Se + Sp −1) + (1−Sp) (Vecchio, 1966). An IF equal to 1.00 indicates that the study coverage generated by the survey question is equal to the true coverage. An IF between 0.75 and 1.25 indicates low population bias. Additionally, for each indicator, the measured coverage values (Pr) were plotted across a range of true coverage (*P*) values. This is done to illustrate whether the survey measure over or under‐estimates the true coverage, given the value of the true coverage.

As a sensitivity analysis, the validation analyses were rerun in the subcohort of women who did not report receiving advice or services from another health provider regarding nutrition for their pregnancy between ANC observations. The follow‐up questionnaire did not ask explicitly about what type of advice or about deworming receipt or weight measurement between observations. This sensitivity analysis was run in this smaller cohort because we are more confident that we observed all care, which serves as a truer gold standard.

Bivariable and multivariable log‐binomial regressions were run to assess whether there were maternal characteristics associated with accurate responses. The binary variable of ‘accuracy’ for each indicator was coded as ‘accurate’ for a maternal response in agreement with the direct observation (true positives and true negatives) versus ‘inaccurate’ those in disagreement (false positives and false negatives). The maternal characteristics included in the models were maternal age, maternal education, whether the woman had a previous live birth (none vs. one or more), and socioeconomic quartile. A socioeconomic composite score was calculated using 11 variables including the number of rooms, fuel source, water source, latrine and a series of ownership variables (examples include cell phones, goats and motorcycles). To account for missing responses, a proportion was calculated where the numerator was the total composite score and the denominator was the number of the 11 questions answered. The proportion was then divided into quartiles. The number of months (continuous variable) since the woman's last ANC observation was included in the model as a measure of recall time. Ethnic group was considered, but all but one enrolled woman was Madeshi so this was dropped due to lack of variation.

The overall validation study sample size was an estimated 300 pregnant women, assuming a 50% prevalence for iron‐folic acid supplementation, to establish a 0.13 wide 95% confidence interval for an AUC equal to 0.50. To allow for loss‐to‐follow up, visits for ANC care that were not observed, and adverse birth outcomes, the study aimed to enrol 450 women total. All analyses were conducted using Stata Version 14.

## 3 RESULTS

**Figure 1:**
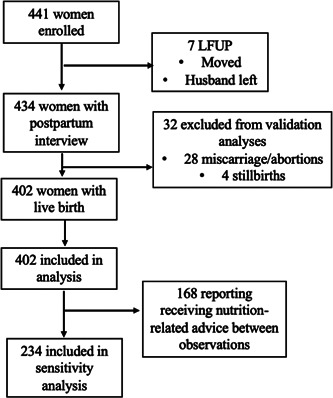
Participant flow chart

Of the 441 women enrolled in the study, 434 women completed the post‐partum interview. Among these, 168 (38.7%) reported receiving nutrition‐related counselling at some point during pregnancy outside of the five study clinics (Figure 1).

Table 1 presents characteristics of enroled women by those who did and did not report receiving care between visits. Nearly 60% of participants reported zero years of education and one‐third of women reported zero prior live births. Compared to those who did not, women who received nutrition‐related counselling between visits attended more observed ANC visits, enrolled earlier in pregnancy, had some formal education, and were more likely to have a prior live birth.

**Table 1:** Characteristics of participants with post‐partum interviews (*N* = 434)

	Did not receive nutrition advice between observations (*N* = 263)		Received nutrition advice between observations (*N* = 171)			Total (*N* = 434)	
Characteristic	Mean (SD)	Range	Mean (SD)	Range	Two sample *t* test *p* value	Mean (SD)	Range
Woman's age, years	22.6 (4.1)	16–41	22.3 (4.3)	16–36	0.437	22.5 (4.2)	16–41
Total number of ANC visits observed	3.7 (2.4)	1–13	5.6 (2.2)	2–14	*p* < .01	4.5 (2.5)	1–14
Number of months between last ANC observation and post‐partum interview	11.0 (3.3)	3–22	9.2 (2.6)	3–17	*p* < .01	10.3 (3.2)	3–22

	Observed all nutrition counselling receipt (*N* = 263)		Received nutrition advice between observations (*N* = 171)		*χ* ^2^ *p* value	Total (*N* = 434)
Most recent pregnancy outcome						
Miscarriage/abortion	26 (9.9%)		2 (1.2%)		*p* < 0.01	28 (6.5%)
Stillbirth	3 (1.1%)		1 (0.6%)			4 (0.9%)
At least one live birth	234 (89.0%)		168 (98.2%)			402 (92.6%)
Four quantiles of SES						
1	111 (42.2%)		56 (32.7%)		0.15	167 (38.5%)
2	46 (17.5%)		28 (16.4%)			74 (17.1%)
3	71 (27.0%)		61 (35.7%)			132 (30.4%)
4	35 (13.3%)		26 (15.2%)			61 (14.0%)
Did this woman have any prior live births?						
Yes	191 (72.6%)		107 (62.6%)		*p* < .05	298 (68.7%)
No	72 (27.4%)		64 (37.4%)			136 (31.3%)
Had the woman receive any schooling?						
No	175 (66.5%)		84 (49.1%)		*p* < .01	259 (59.7%)
Yes	88 (33.5%)		87 (50.9%)			175 (40.3%)
Trimester at enrolment						
1–3 months	102 (38.8%)		88 (51.5%)		*p* < .01	190 (43.8%)
4–6 months	153 (58.2%)		83 (48.5%)			236 (54.4%)
7–9 months	8 (3.0%)		0 (0.0%)			8 (1.8%)

Abbreviation: ANC, antenatal care.

The observed and reported coverage of the indicators of interest is presented in Table 2. For all but the two weight‐related indicators, the coverage measured by the maternal report was greater than the coverage determined by the direct observation of ANC. Very few women responded ‘don't know’ at the post‐partum interview for any indicator. At the post‐partum interview, 11% of the women received ‘a little help’ answering the survey questions from their mothers‐in‐law and 2% received ‘a little help’ from their husbands. No women were recorded receiving ‘a lot of help’ answering the questions.

**Table 2:** Observed and reported coverage of indicators assessed (*N* = 10)

		Post‐partum	
At any point in pregnancy, the woman…	During pregnancy Observed at ANC	Maternal report	# reporting ‘Don't know’
Nutrition‐related services			
Received deworming	77.90%	85.80%	1
Received or was told to buy calcium	89.80%	90.40%	1
Had weight measured	99.50%	97.50%	2
Nutrition‐related counselling			
Was counselled on weight	99.20%	93.50%	3
Was counselled on nutrition, generally	95.50%	98.80%	0
Was counselled on eating more food	91.40%	96.50%	0
Was counselled on diverse diet	95.00%	99.70%	0
Was counselled on managing nausea/vomiting	24.70%	52.40%	0
Was counselled on not drinking alcohol	41.60%	72.60%	1
Was counselled on not smoking tobacco/using pan	50.40%	72.60%	1

Abbreviation: ANC, antenatal care.

The indicator validation results are presented in Table 3. There were no indicators that had both high individual‐level validity (AUC > 0.70) and low population bias (0.75 < IF < 1.25). The majority of indicators had high sensitivity (Se > 90%), but very poor specificity, meaning that women who were not observed receiving a service reported receiving it. Comparing Tables 2 and 3, those indicators with higher true coverage estimates had higher sensitivity and lower specificity estimates than indicators with lower true coverage. For five of the indicators (weight measurement, counselling on weight, general nutrition counselling, counselling on eating more food and counselling on a diverse diet) there was a high uncertainty due to the low number of true positives or negatives and should be interpreted cautiously. Seven of the 10 indicators had low population bias, primarily driven by the high coverage and sensitivity. For the other three indicators, the survey questions resulted in an overestimation of the true coverage.

**Table 3:** Validation results for indicators assessed (*N* = 10)

Indicator	Sensitivity (%) (95% CI)	Specificity (%) (95% CI)	AUC	Survey coverage (%) based on Se and Sp	IF
Nutrition‐related services					
Received deworming	90.4 (86.6–93.4)	30.7 (21.3–41.4)	0.61 (0.55–0.66)	85.8%	1.10
Received or was told to buy calcium	92.1 (88.8–94.7)	25.0 (12.7–41.2)	0.59 (0.52–0.66)	90.4%	1.01
Had weight measured	97.5 (95.4–98.8)	0.0* (0.0–84.2)	0.49* (0.48–0.50)	97.5%	0.98
Nutrition‐related counselling					
Was counselled on weight	93.8 (90.8–96.0)	33.3* (0.8–90.6)	0.64* (0.31–0.96)	93.6%	0.94
Was counselled on nutrition, generally	98.7 (97.0–99.6)	0.0* (0.0–18.5)	0.49*(0.49–0.50)	98.8%	1.03
Was counselled on eating more food	96.4 (94.0–98.1)	2.9* (0.1–15.3)	0.50* (0.47–0.53)	91.1%	1.00
Was counselled on diverse diet	99.7 (98.5–100.0)	0.0* (0.0–16.8)	0.50*(0.50–0.50)	99.7%	1.05
Was counselled on managing nausea/vomiting	57.1 (46.7–67.1)	49.2 (43.4–55.0)	0.53 (0.47–0.59)	52.4%	2.12
Was counselled on not drinking alcohol	83.8 (77.4–89.1)	35.5 (29.3–42.0)	0.60 (0.55–0.64)	72.5%	1.74
Was counselled on not smoking tobacco/using paan	82.2 (76.2–87.2)	37.2 (30.5–44.3)	0.60 (0.55–0.64)	72.6%	1.44

Abbreviations: AUC, area under the operating curve; IF, inflation factor; Se, sensitivity; Sp, specificity.

* Indicates uncertainty around this point estimate, as a small number of true positives or negatives resulted in an estimate with a 95% confidence interval greater than 15%.

Graphs were created which plotted the IF across all possible coverage values (Figure S1a–e). The indicators with near‐complete coverage and specificity values equal to zero were not plotted. At lower coverage levels, all indicators would drastically overestimate the true coverage. The deworming receipt, calcium and weight counselling indicators perform better at high coverage levels (>80% true coverage), resulting in only a slight over or underestimation. The remaining three indicators, counselling on nausea management and tobacco and alcohol use, do not perform well across the spectrum of true coverage levels.

The sensitivity analysis among pregnant women who reported never receiving nutrition advice between ANC observations is presented in Table S1. All women reported receiving information about a diverse diet, so a 2 × 2 table could not be constructed for this indicator. The restriction improved specificity in some cases, for example for the deworming indicator specificity, increased from 30.7% to 41.8%. However, for the majority of indicators changes to the summary level AUC and IF values did not change by more than a percentage point.

None of the maternal characteristics was associated with the accuracy of reporting for any indicator (Table 4). Maternal age, education and having a previous live birth did not have a consistent direction of association with accuracy, nor were the magnitudes of association large or statistically significant. Compared to the lowest quartile, the higher quartiles were associated with slightly more accuracy for the 10 indicators, although this association was not statistically significant for any relationship. Finally, there was no association between time between the last ANC observation and the post‐partum interview, measured in months and accuracy.

**Table 4:** Maternal characteristics associated with accuracy

Indicator	Accurate response %	Maternal age ARR (95% CI)	Any education ARR (95% CI)	No previous live birth ARR (95% CI)	Socioeconomic Status (ref:First quartile)	Second quartile ARR (95% CI)	Third quartile ARR (95% CI)	Fourth quartile ARR (95% CI)	Time since last ANC observation (in months) ARR (95% CI)
Deworming	77.10%	0.99 (0.98– 1.03)	0.94 (0.73–1.22)	0.92 (0.69–1.23)		1.19 (0.86–1.64)	1.14 (0.86–1.52)	1.18 (0.81–1.71)	1.00 (0.97–1.04)
Receipt of Ca or Ca info	84.10%	1.00 (0.97–1.03)	1.09 (0.85–1.38)	1.01 (0.77–1.32)		1.02 (0.74–1.47)	1.12 (0.85–1.47)	1.09 (0.77–1.55)	0.97 (0.94–1.01)
Weight measurement	96.50%	1.00 (0.97–1.03)	1.03 (0.82–1.29)	0.99 (0.77–1.28)		0.99 (0.75–1.33)	1.01 (0.79–1.31)	1.02 (0.73–1.42)	0.99 (0.96–1.03)
Counselled on…									
Weight	89.80%	1.00 (0.98–1.03)	1.01 (0.80–1.28)	0.98 (0.75–1.27)		1.01 (0.75–1.37)	1.06 (0.81–1.38)	1.06 (0.75–1.50)	0.99 (0.96–1.03)
Nutrition, generally	94.30%	1.00 (0.97–1.03)	1.00 (0.79–1.25)	1.03 (0.79–1.33)		1.07 (0.80–1.43)	1.05 (0.82–1.36)	1.06 (0.76–1.49)	0.99 (0.96–1.03)
Eating more	87.30%	0.99 (0.96–1.02)	0.97 (0.76–1.23)	1.01 (0.77–1.32)		1.02 (0.75–1.38)	1.03 (0.79–1.34)	1.08 (0.76–1.53)	0.98 (0.94–1.01)
Diverse diet	93.50%	0.99 (0.97–1.02)	1.00 (0.79–1.25)	1.01 (0.78–1.30)		1.08 (0.80–1.44)	1.05 (0.81–1.36)	1.05 (0.75–1.47)	0.99 (0.96–1.03)
Managing nausea	50.50%	1.01 (0.99–1.04)	0.89 (0.71–1.11)	1.05 (0.82–1.35)		1.07 (0.80–1.42)	1.16 (0.92–1.147)	1.03 (0.73–1.44)	0.98 (0.95–1.02)
Not drinking alcohol	55.40%	1.00 (0.98–1.03)	1.16 (0.95–1.41)	1.16 (0.93–1.45)		1.02 (0.77–1.36)	1.12 (0.88–1.41)	1.17 (0.89–1.55)	1.01 (0.98–1.04)
Not smoking tobacco/using paan	59.70%	1.00 (0.98–1.03)	1.17 (0.98–1.38)	1.08 (0.88–1.33)		1.13 (0.88–1.46)	1.22 (0.99–1.51)	1.23 (0.95–1.58)	0.99 (0.97–1.02)

Abbreviations: ANC, antenatal care; ARR, adjusted risk ratio.

## 4 DISCUSSION

This study assessed the validity of maternal recall for 10 nutrition‐related interventions provided during ANC in rural, Southern Nepal; none of the indicators performed well for both individual‐level validity and population‐level bias. For individual‐level validity, the indicators' performances ranged from equal to a random guess to moderately well. None were assessed to have high individual level validity. In similar populations with high true coverage, 7 of the 10 indicators (deworming receipt, calcium receipt or instructions to buy, weight measurement, counselling on weight, general nutrition counselling, counselling on eating more food and counselling on a diverse diet) demonstrated low population bias and would produce accurate survey coverage measurements. The other three indicators (counselling on nausea/vomiting management, counselling on not drinking, counselling on not smoking), which had lower true coverage measures, demonstrated high population bias resulting in a survey measurement that greatly overestimated the true measurement.

It is important to consider how the observation by our study staff of counselling being delivered by a provider does not necessarily mean that the information was absorbed and understood by the pregnant woman. A recent review highlights the gaps in counselling quality in Southeast Asia, where short duration and low frequency of counselling contacts as well as gaps in health worker training posed problems for successful information transfer (Torlesse et al., 2021). Within the counselling topics examined in our study, the sensitivity of the nutrition‐specific counselling was higher than that of nausea management or substance use, indicating that women who were observed receiving nutrition‐related counselling also recalled receiving this information, likely indicating a successful information transfer. The nutrition counselling was often communicated with a visual aid, for example, a flip chart, which may have resulted in more effective communication than nausea or substance use counselling (Odackal et al., 2020).

The indicators with higher true coverage values were found to have had high sensitivity and lower specificity in our study population. The authors of another study in the same area found a similar relationship, concluding that the high coverage of service may result in a woman reporting receipt because she assumed she should have received it, rather than actually recalling its receipt (Carter et al., 2021).

The validity of maternal recall of weight measurement has been examined in two prior studies. The study in rural China, which compared maternal reports 2–5 years after delivery to paper and electronic‐based health records, also had a very high prevalence of weight measurement during pregnancy (98%) and reported findings nearly identical to ours; sensitivity equal to 0.98, a specificity of 0.0 and an AUC equal to 0.49 (Liu et al., 2013). The similarity between the two sets of findings is likely driven by the near‐complete coverage in both populations. With almost complete coverage, the specificity is based on so few observations and a small number of false positives can drive it downward. The analysis of a three‐country survey reported better individual‐level validity for weight measurement than in our study (McCarthy et al., 2020). However, these results compared maternal reports at exit interviews to direct observation, whereas the recall period in our study was approximately 6‐months post‐partum and on average 10 months following a woman's last ANC visit. The three‐country survey assessed recall of advice on diet and nutrition in a Bangladeshi population, where women were able to more accurately recall immediately following a visit compared to our longer post‐partum recall period (McCarthy et al., 2020). However, we did not find an association between the length of the recall period and accuracy.

The three‐country validation study that compared direct observation of antenatal and post‐natal care services to exit interviews reported that women were better able to recall concrete interventions, such as blood pressure measurements, than topics discussed in counselling (McCarthy et al., 2020). For the indicators with adequate confidence intervals assessed in our study, comparing the concrete intervention, received deworming medication, to the counselling topics, management of nausea and substance use, the differences are not as apparent. The sensitivity of receipt of deworming medication is greater than the counselling topic indicators, but its lower specificity results in an AUC nearly equal to that of both substance use indicators. The higher sensitivity could be due to a number of factors. Women may be able to recall the concrete indicator better than the counselling, as McCarthy et al. posited. The true coverage of deworming was greater than that of the counselling topics, which could lead women to more frequently report its receipt as described earlier (Carter et al., 2021). This hypothesis would also help explain the lower specificity of the deworming medication indicator, where women report receiving the indicator because they presume to have received it instead of actually recalling its receipt.

After marriage in Nepal, women typically move in with their husband's families where the mothers‐in‐law preside over many of the household decisions. For first pregnancies, however, some women return to their familial home or *maiti*. Previous studies in Nepal have demonstrated that mothers‐in‐law have particular influence over antenatal and perinatal care decisions (Masvie, 2006; Simkhada et al., 2010). Anecdotally, many of the women in our population attended ANC with their mother or mothers‐in‐law, which could have had an impact on the pregnant woman's intake of information and resulted in lower recall accuracy. It is possible that the mothers‐in‐law engaged with the provider during the counselling session more than the pregnant woman herself, which could reduce the pregnant woman's ability to recall receiving this information post‐partum. Unfortunately, we did not record who else was present at the ANC visits. We did measure whether the woman received help answering the questions at the post‐partum interview, though when included in the model (data not shown) this factor had no association with accuracy or impact on the other included variables. Additionally, whether the mother‐in‐law helped answer questions during the post‐partum interview does not necessarily reflect their presence or role at the ANC visit. For future research, it would be interesting to examine whether the presence of specific family members during ANC has an impact on the accuracy of maternal reports.

A sensitivity analysis that was limited to women who never reported receiving nutrition‐related advice elsewhere than where the study observed them did not improve validity for the majority of indicators. However, even with this restriction, we may not have produced a true gold standard; we asked about receiving advice from a health care provider, but this does not limit the inclusion of advice from peers, mothers or mothers‐in‐law. Therefore, it is possible that specificity was not improved because although the women in the subset did not receive advice from health providers, they may be recalling information received from other sources that we did not capture in our data collection. Furthermore, we were unable to restrict the analysis to receipt of the other nutrition services like deworming and weight measurement between visits because these were not included in the follow‐up questionnaire.

In the study population, nearly all women received counselling on nutrition (diversity of diet and increasing intake) at some point during pregnancy. However, the true coverage of counselling on nausea management and not drinking or smoking was much lower. This may be because counselling on these topics is not standard for all women, rather just women who complain of nausea or who report drinking or smoking. The low specificity for these indicators could also be explained by the fact that women could have picked up this information from peers, mothers or mothers‐in‐law, or it may just be considered general knowledge. The proportion of women who reported receiving these counselling messages was 20–30 absolute percentage points higher than the observed proportion. Therefore, although the question in the survey asked about receiving this information at our health posts specifically, the women may inherently know the information or have heard it elsewhere and thus reported receiving it during ANC.

There were no maternal characteristics that had a statistically significant relationship with accurate maternal reports. This finding is consistent with some studies (Carter et al., 2018; McCarthy et al., 2018) but another examining accuracy of recall of low birth weight at the same study site found that higher education and parity were associated with the accurate maternal recall (Chang et al., 2018). The number of months that had passed since the last ANC observation also had no association with maternal accuracy, which was the same finding in the low birth weight study in Nepal (Chang et al., 2018). Our range of recall time is much shorter than the three‐year range of the DHS and, therefore, may not be extrapolated to the longer recall period of the DHS. However, a 6‐month follow‐up period was feasible resource‐wise and much more reflective of a household survey than other studies that use the exit interview to collect maternal recall.

A strength of this study is that we employed direct observation by a trained study observer as the gold standard. The study observers went through detailed training and had to achieve a standard for intra‐ and interobserver reliability before being approved for the work. A second strength was the length of the recall period, which was more similar to the DHS and MICS recall periods of 2–5 years than other studies that have compared maternal reports at an exit interview. A limitation of the study was that it only captured women attending ANC at government health posts, so the findings may not be generalisable to women who attended private health facilities only or who did not attend ANC. Although we did our best to account for care‐seeking outside of the study observations, a final limitation is that we were unable to observe care at every possible source in the community over the entire pregnancy.

Large household surveys like the DHS are the main source of coverage data, but these findings suggest that accuracy data produced by maternal recall in these surveys may be variable. The efforts to strengthen electronic health records and information systems in these settings could offer an alternate measurement method, however, they do not currently capture counselling provision during ANC. Updating these systems to include counselling measurement should be a component in future efforts to strengthen national health systems. Routine health data could be used in conjunction with data generated by household surveys to best inform future counselling coverage measurement.

## 5 CONCLUSION

This study adds to the growing evidence base demonstrating that there is variability in how accurately a woman can recall services received during ANC. The measurement of the 10 indicators by the maternal report had low to moderate individual‐level validity and low to high population‐level bias. The high coverage of five of the 10 indicators limited our certainty surrounding these estimates and they should be examined in additional settings across a range of true coverage.

## CONFLICT OF INTERESTS

The authors declare that there are no conflict of interests.

## AUTHOR CONTRIBUTIONS

MM and JK designed the study. TPL, SKK, SL, JK and EB contributed to the implementation of the study. EB conducted analyses and wrote the first draft of the manuscript. MM, RH and JK submitted edits to the manuscript. MM, JK, TPL, SKK and SL all have read and approved the final version of the manuscript.

## Supporting information

Supporting information.

## Data Availability

The data that support the findings of this study are available from the corresponding author upon reasonable request.

## References

[mcn13303-bib-0001] Abraham, M. , Alramadhan, S. , Iniguez, C. , Duijts, L. , Jaddoe, V. W. V. , Den Dekker, H. T. , Crozier, S. , Godfrey, K. M. , Hindmarsh, P. , Vik, T. , Jacobsen, G. W. , Hanke, W. , Sobala, W. , Devereux, G. , & Turner, S. (2017). A systematic review of maternal smoking during pregnancy and fetal measurements with meta‐analysis. PLOS One, 12(2), e0170946. 28231292 10.1371/journal.pone.0170946 PMC5322900

[mcn13303-bib-0002] Adhikari, K. , Liabsuetrakul, T. , & Pradhan, N. (2009). Effect of education and pill count on hemoglobin status during prenatal care in Nepalese women: A randomized controlled trial. The Journal of Obstetrics and Gynaecology Research, 35(3), 459–466. 19527383 10.1111/j.1447-0756.2008.00970.x

[mcn13303-bib-0003] Bhutta, Z. A. , Ahmed, T. , Black, R. E. , Cousens, S. , Dewey, K. , Giugliani, E. , Haider, B. A. , Kirkwood, B. , Morris, S. S. , Sachdev, H. , & Shekar, M. (2008). What works? Interventions for maternal and child undernutrition and survival. Lancet (London, England), 371(9610), 417–440. 10.1016/S0140-6736(07)61693-6 18206226

[mcn13303-bib-0004] Bhutta, Z. A. , Das, J. K. , Rizvi, A. , Gaffey, M. F. , Walker, N. , Horton, S. , Webb, P. , Lartey, A. , Black, R. E. , & Lancet Nutrition Interventions Review Group, the Maternal and Child Nutrition Study, G. (2013). Evidence‐based interventions for improvement of maternal and child nutrition: What can be done and at what cost? Lancet (London, England), 382(9890), 452–477. 10.1016/S0140-6736(13)60996-4 23746776

[mcn13303-bib-0005] Black, R. E. , Allen, L. H. , Bhutta, Z. A. , Caulfield, L. E. , de Onis, M. , Ezzati, M. , Mathers, C. , Rivera, J. , & Maternal and Child Undernutrition Study, G. (2008). Maternal and child undernutrition: Global and regional exposures and health consequences. Lancet (London, England), 371(9608), 243–260. 10.1016/S0140-6736(07)61690-0 18207566

[mcn13303-bib-0006] Black, R. E. , Victora, C. G. , Walker, S. P. , Bhutta, Z. A. , Christian, P. , de Onis, M. , Ezzati, M. , Grantham‐McGregor, S. , Katz, J. , Martorell, R. , Uauy, R. , & Maternal and Child Nutrition Study, G. (2013). Maternal and child undernutrition and overweight in low‐income and middle‐income countries. Lancet (London, England), 382(9890), 427–451. 10.1016/S0140-6736(13)60937-X 23746772

[mcn13303-bib-0007] Blanc, A. K. , Warren, C. , McCarthy, K. J. , Kimani, J. , Ndwiga, C. , & RamaRao, S. (2016). Assessing the validity of indicators of the quality of maternal and newborn health care in Kenya. Journal of Global Health, 6(1), 010405. 27231541 10.7189/jogh.06.010405 PMC4871064

[mcn13303-bib-0008] Bryce, E. , Munos, M. , Lama, T. , Khatry, S. K. , LeClerq, S. , & Katz, J. (2021). Validation of maternal report of receipt of iron folic‐acid supplementation during antenatal care in rural southern Nepal. Journal of Nutrition. 10.1093/jn/nxab336 PMC8754516 34549300

[mcn13303-bib-0009] Carter, E. D. , Chang, K. T. , Mullany, L. C. , Khatry, S. K. , LeClerq, S. C. , Munos, M. K. , & Katz, J. (2021). Reliability of maternal recall of delivery and immediate newborn care indicators in Sarlahi, Nepal. BMC Pregnancy and Childbirth, 21(1), 82. 33494712 10.1186/s12884-021-03547-5 PMC7831166

[mcn13303-bib-0010] Carter, E. D. , Ndhlovu, M. , Munos, M. , Nkhama, E. , Katz, J. , & Eisele, T. P. (2018). Validity of maternal report of care‐seeking for childhood illness. Journal of Global Health, 8(1), 010602. 29619212 10.7189/jogh.08.010602 PMC5854307

[mcn13303-bib-0011] Chang, K. T. , Mullany, L. C. , Khatry, S. K. , LeClerq, S. C. , Munos, M. K. , & Katz, J. (2018). Validation of maternal reports for low birthweight and preterm birth indicators in rural Nepal. Journal of Global Health, 8(1), 010604. 29899981 10.7189/jogh.08.010604 PMC5997365

[mcn13303-bib-0012] Christian, P. , Katz, J. , Wu, L. , Kimbrough‐Pradhan, E. , Khatry, S. K. , LeClerq, S. C. , & West KP, Jr . (2008). Risk factors for pregnancy‐related mortality: A prospective study in rural Nepal. Public Health, 122(2), 161–172. 17826810 10.1016/j.puhe.2007.06.003 PMC2367232

[mcn13303-bib-0013] Demilew, Y. M. , Alene, G. D. , & Belachew, T. (2020). Effects of guided counseling during pregnancy on birth weight of newborns in West Gojjam Zone, Ethiopia: A cluster‐randomized controlled trial. BMC pediatrics, 20(1), 466. 33023521 10.1186/s12887-020-02363-8 PMC7542400

[mcn13303-bib-0014] Girard, A. W. , & Olude, O. (2012). Nutrition education and counselling provided during pregnancy: Effects on maternal, neonatal and child health outcomes. Paediatric and Perinatal Epidemiology, 26(Suppl 1), 191–204. 22742611 10.1111/j.1365-3016.2012.01278.x

[mcn13303-bib-0015] Goldstein, R. F. , Abell, S. K. , Ranasinha, S. , Misso, M. , Boyle, J. A. , Black, M. H. , Li, N. , Hu, G. , Corrado, F. , Rode, L. , Kim, Y. J. , Haugen, M. , Song, W. O. , Kim, M. H. , Bogaerts, A. , Devlieger, R. , Chung, J. H. , & Teede, H. J. (2017). Association of gestational weight gain with maternal and infant outcomes: A systematic review and meta‐analysis. Journal of the American Medical Association, 317(21), 2207–2225. 28586887 10.1001/jama.2017.3635 PMC5815056

[mcn13303-bib-0016] Han, Z. , Lutsiv, O. , Mulla, S. , Rosen, A. , Beyene, J. , McDonald, S. D. , & Knowledge Synthesis, G. (2011). Low gestational weight gain and the risk of preterm birth and low birthweight: A systematic review and meta‐analyses. Acta Obstetricia et Gynecologica Scandinavica, 90(9), 935–954. 21623738 10.1111/j.1600-0412.2011.01185.x

[mcn13303-bib-0017] Han, Z. , Mulla, S. , Beyene, J. , Liao, G. , & McDonald, S. D. (2011). Knowledge Synthesis G. Maternal underweight and the risk of preterm birth and low birth weight: A systematic review and meta‐analyses. International Journal of Epidemiology, 40(1), 65–101. 21097954 10.1093/ije/dyq195

[mcn13303-bib-0018] Hofmeyr, G. J. , Lawrie, T. A. , Atallah, A. N. , & Torloni, M. R. (2018). Calcium supplementation during pregnancy for preventing hypertensive disorders and related problems. The Cochrane Database of Systematic Reviews, 10, CD001059. 30277579 10.1002/14651858.CD001059.pub5 PMC6517256

[mcn13303-bib-0019] Liu, L. , Li, M. , Yang, L. , Ju, L. , Tan, B. , Walker, N. , Bryce, J. , Campbell, H. , Black, R. E. , & Guo, Y. (2013). Measuring coverage in MNCH: A validation study linking population survey derived coverage to maternal, newborn, and child health care records in rural China. PLOS One, 8(5), e60762. 23667429 10.1371/journal.pone.0060762 PMC3646215

[mcn13303-bib-0020] Masvie, H. (2006). The role of Tamang mothers‐in‐law in promoting breast feeding in Makwanpur District, Nepal. Midwifery, 22(1), 23–31. 15967547 10.1016/j.midw.2005.02.003

[mcn13303-bib-0021] McCarthy, K. J. , Blanc, A. K. , Warren, C. , Bajracharya, A. , & Bellows, B. (2020). Validating women's reports of antenatal and postnatal care received in Bangladesh, Cambodia and Kenya. BMJ Global Health, 5, 5.

[mcn13303-bib-0022] McCarthy, K. J. , Blanc, A. K. , Warren, C. E. , Kimani, J. , Mdawida, B. , & Ndwidga, C. (2016). Can surveys of women accurately track indicators of maternal and newborn care? A validity and reliability study in Kenya. Journal of Global Health, 6(2), 020502. 27606061 10.7189/jogh.06.020502 PMC5012235

[mcn13303-bib-0023] McCarthy, K. J. , Blanc, A. K. , Warren, C. E. , & Mdawida, B. (2018). Women's recall of maternal and newborn interventions received in the postnatal period: A validity study in Kenya and Swaziland. Journal of Global Health, 8(1), 010605. 29904605 10.7189/jogh.08.010605 PMC5983915

[mcn13303-bib-0024] Munos, M. K. , Blanc, A. K. , Carter, E. D. , Eisele, T. P. , Gesuale, S. , Katz, J. , Marchant, T. , Stanton, C. K. , Campbell, H. , & Improving Coverage Measurement, G. (2018). Validation studies for population‐based intervention coverage indicators: Design, analysis, and interpretation. Journal of Global Health, 8(2), 020804. 30202519 10.7189/jogh.08.020804 PMC6126515

[mcn13303-bib-0025] Nepal MoH . (2017). Nepal Demographic and Health Survey 2016. Ministry of Health, Nepal.

[mcn13303-bib-0026] Nikièma, L. , Huybregts, L. , Martin‐Prevel, Y. , Donnen, P. , Lanou, H. , Grosemans, J. , Offoh, P. , Dramaix‐Wilmet, M. , Sondo, B. , Roberfroid, D. , & Kolsteren, P. (2017). Effectiveness of facility‐based personalized maternal nutrition counseling in improving child growth and morbidity up to 18 months: A cluster‐randomized controlled trial in rural Burkina Faso. PLOS One, 12(5), e0177839. 28542391 10.1371/journal.pone.0177839 PMC5444625

[mcn13303-bib-0027] Nykjaer, C. , Alwan, N. A. , Greenwood, D. C. , Simpson, N. A. , Hay, A. W. , White, K. L. , & Cade, J. E. (2014). Maternal alcohol intake prior to and during pregnancy and risk of adverse birth outcomes: Evidence from a British cohort. Journal of Epidemiology and Community Health, 68(6), 542–549. 24616351 10.1136/jech-2013-202934 PMC4033207

[mcn13303-bib-0028] Odackal, N. J. , Conaway, M. , Cha, J. , & Swanson, J. R. (2020). Prenatal consults with illustrated literature (PnCIL): A RCT studying visual aids during prenatal consults. Journal of Perinatology, 40(8), 1154–1162. 32514007 10.1038/s41372-020-0709-y

[mcn13303-bib-0029] Ota, E. , Hori, H. , Mori, R. , Tobe‐Gai, R. , & Farrar, D. (2015). Antenatal dietary education and supplementation to increase energy and protein intake. The Cochrane Database of Systematic Reviews, 6, CD000032. 10.1002/14651858.CD000032.pub3 PMC12634316 26031211

[mcn13303-bib-0030] Salam, R. , Cousens, S. , Welch, V. , Gaffey, M. , Middleton, P. , Makrides, M. , Arora, P. , & Bhutta, Z. A. (2019). Mass deworming for soil‐transmitted helminths and schistosomiasis among pregnant women: A systematic review and individual participant data meta‐analysis. Campbell Systematic Reviews, 15, 3. 10.1002/cl2.1052 PMC8356523 37131518

[mcn13303-bib-0031] Say, L. , Chou, D. , Gemmill, A. , Tunçalp, Ö. , Moller, A. B. , Daniels, J. , Gülmezoglu, A. M. , Temmerman, M. , & Alkema, L. (2014). Global causes of maternal death: A WHO systematic analysis. Lancet Globalization and Health, 2(6), e323–e333. 10.1016/S2214-109X(14)70227-X 25103301

[mcn13303-bib-0032] Simkhada, B. , Porter, M. A. , & van Teijlingen, E. R. (2010). The role of mothers‐in‐law in antenatal care decision‐making in Nepal: A qualitative study. BMC Pregnancy and Childbirth, 10, 34. 20594340 10.1186/1471-2393-10-34 PMC2910658

[mcn13303-bib-0033] Stanton, C. K. , Rawlins, B. , Drake, M. , Dos Anjos, M. , Cantor, D. , Chongo, L. , Chavane, L. , da Luz Vaz, M. , & Ricca, J. (2013). Measuring coverage in MNCH: Testing the validity of women's self‐report of key maternal and newborn health interventions during the peripartum period in Mozambique. PLOS One, 8(5), e60694. 23667427 10.1371/journal.pone.0060694 PMC3646219

[mcn13303-bib-0034] The DHS Program . (2021). *Tableau family planning dashboard*. https://dhsprogram.com/

[mcn13303-bib-0035] Torlesse, H. , Benedict, R. K. , Craig, H. C. , & Stoltzfus, R. J. (2021). The quality of maternal nutrition and infant feeding counselling during antenatal care in South Asia. Maternal & child nutrition, 17, e13153. 33554434 10.1111/mcn.13153 PMC8189234

[mcn13303-bib-0036] Vecchio, T. J. (1966). Predictive value of a single diagnostic test in unselected populations. The New England Journal of Medicine, 274(21), 1171–1173. 5934954 10.1056/NEJM196605262742104

[mcn13303-bib-0037] WHO . (2016). WHO recommendations on antenatal care for a positive pregnancy experience. Geneva, Switzerland. 28079998

