# Supplementary material for: Validation of maternal report of nutrition‐related interventions and counselling during antenatal care in southern Nepal
Source: Matern Child Nutr. 2021 Dec 14;18(2):e13303. doi: 10.1111/mcn.13303 (PMC8932699; doi:10.1111/mcn.13303)
Supplement: Supplementary file 1 — Supporting information. [file MCN-18-e13303-s001.docx]

**Supplementary figures 1a-1e. Measured versus true coverage for indicators of nutrition-related services**

**1a**

**1b**

**1c**

**1d**

**1e**

**Supplementary Table 1. Sensitivity analysis in sub-cohort of women**

| Indicator | Sensitivity (%)  (95% CI) | Specificity (%)  (95% CI) | AUC | Survey coverage (%) based on Se & Sp | IF |
| --- | --- | --- | --- | --- | --- |
| Nutrition-related services |  |  |  |  |  |
| Received deworming | 89.9  (84.6 to 93.9) | 41.8*  (28.7 to 55.9) | 0.66*  (0.59 to 0.73) | 82.5% | 1.08 |
| Received or was told to buy calcium | 89.0  (83.8 to 93.0) | 24.1*  (10.3 to 43.5) | 0.57*  (0.48 to 0.65) | 87.3% | 1.00 |
| Had weight measured | 97.0  (93.8 to 98.8) | 0.0*  (0.0 to 84.2) | 0.48*  (0.47 to 0.50) | 97.0% | 0.98 |
| Nutrition-related counseling |  |  |  |  |  |
| Was counseled on weight | 91.8  (87.4 to 95.1) | 33.3*  (0.8 to 90.6) | 0.63*  (0.30 to 0.95) | 91.5% | 0.93 |
| Was counseled on nutrition, generally | 98.2  (95.3 to 99.5) | 0.0*  (0.0 to 19.5) | 0.49*  (0.48 to 0.50) | 98.3% | 1.06 |
| Was counseled on eating more food | 94.1  (89.9 to 96.9) | 3.7*  (0.1 to 19.0) | 0.49*  (0.45 to 0.53) | 94.4% | 1.07 |
| Was counseled on diverse diet | -- | -- | -- | -- | -- |
| Was counseled on managing nausea/ vomiting | 46.7*  (31.7 to 62.1) | 48.6  (41.2 to 56.1) | 0.48*  (0.39 to 0.56) | 50.5% | 2.58 |
| Was counseled on not drinking alcohol | 86.3  (77.7 to 92.5) | 38.8*  (30.7 to 47.5) | 0.63*  (0.57 to 0.68) | 71.4% | 1.76 |
| Was counseled on not smoking tobacco/using paan | 83.8*  (75.6 to 90.1) | 39.0*  (30.4 to 48.2) | 0.61*  (0.56 to 0.67) | 71.8% | 1.51 |

* Indicates uncertainty around this point estimate, as small number of true positive or negatives resulted in an estimate with a 95% confidence interval greater than fifteen percent.
